# A cross-sectional study of website claims related to diagnoses and treatment of non-musculoskeletal conditions

**DOI:** 10.1186/s12998-020-00305-w

**Published:** 2020-03-30

**Authors:** Rikke Krüger Jensen, Mie Elisa Irgens Agersted, Heike Aaby Nielsen, Søren O’Neill

**Affiliations:** 1 Department of Sport Science and Clinical Biomechanics, University of Southern Denmark, Campusvej 55, 5230 Odense M, Denmark; 2 Nordic Institute of Chiropractic and Clinical Biomechanics, Campusvej 55, 5230 Odense M, Denmark; 3 Private chiropractic practice, Kiropraktisk Center Naestved, Gammel Holstedvej 22, 4700, Naestved, Denmark; 4 Private chiropractic practice, Kiropraktorerne Westloft, Axeltorv 8, 1, 1609 Copenhagen V, Denmark; 5 Spine Centre of Southern Denmark, University Hospital of Southern Denmark, Oestre Hougvej 55, 5500 Middelfart, Denmark; 6 Department of Regional Health Research, University of Southern Denmark, J.B Winsløwsvej 19, 5000 Odense C, Denmark

**Keywords:** Chiropractor, Website, Non-musculoskeletal, Advertising, Type O disorders

## Abstract

**Background:**

The Danish Authorisation Act sets out the chiropractic scope of practice. Under this legislation the scope of practice is diagnostics, prevention and treatment of biomechanical disorders of the spine, pelvis and extremities. Despite this and an international movement toward a scientifically active, evidence-based profession with a focus on treatment of musculoskeletal disorders, a large proportion of chiropractors still offer treatment of non-musculoskeletal disorders.

This study aimed to investigate the content and characteristics of website claims by chiropractors in Denmark on non-musculoskeletal conditions and to assess whether these were aligned with the Danish Authorisation Act of the chiropractic scope of practice.

**Methods:**

In this cross-sectional study data on a representative sample were collected from chiropractic clinic websites in Denmark. Information on non-musculoskeletal conditions from the websites was categorised. For each non-musculoskeletal condition, it was noted whether a clarifying explanation justifying the presence of the diagnosis was available and what it said. These explanations were assessed and categorised according to agreement or disagreement with the chiropractic scope of practice as defined by the Danish Authorisation Act. In addition, data on geographic location, clinic size, reimbursement coverage, country of education and special clinical focus (children, athletes, etc) were collected. Differences in characteristics of the clinics and the frequency of reporting non-musculoskeletal conditions were tested using Pearson’s chi-squared or Fisher’s exact test.

**Results:**

A geographically stratified, random sample of 139 (57%) websites was included from chiropractic clinics in Denmark. In total, 36 (26%) of the sampled websites mentioned conditions of non-musculoskeletal origin that was not accompanied by a clarifying explanation in agreement with the chiropractic scope of practice. A positive association between advertising infant or children’s care and advertising treatment for non-musculoskeletal disorders (without adequate explanation) was observed.

**Conclusions:**

A total of 36 (26%) of the sampled chiropractic websites in Denmark mentioned diagnoses or symptoms of non-musculoskeletal origin on their websites without presenting an adequate clarifying explanation in agreement with the chiropractic scope of practice as defined by the Danish Authorisation Act. This could be misleading for patients seeking care for non-musculoskeletal conditions and consequently lead to inappropriate treatment.

## Background

The historical theory of the ‘vertebral subluxation’ purported that such lesions could affect the nervous system in a deleterious way and cause almost any kind of illness and disease [1]. These concepts were part of the paradigm which formed the chiropractic profession more than a 100 years ago [1]. The theory purported to explain how spinal manipulation could treat musculoskeletal disorders as well as most non-musculoskeletal disorders such as asthma, allergy, high or low blood pressure, irritable bowel disease etc. [2] Early anecdotal evidence even suggested that spinal manipulation was effective in curing deafness [1]. In step with the rapid development in health science, including research into musculoskeletal disorders and spinal manipulation, many chiropractors have moved away from these early theories [3]. In many countries chiropractors have become an integrated part of the established health care systems although the profession is still not regulated in many countries [4, 5].

A 2019 review of the global chiropractic workforce [4] found that in 90 countries, direct access to a chiropractor was available in 90% of the countries. Chiropractic services were partially or fully covered by government and/or private health schemes in 51% countries and legally recognized in 76% of the 90 countries. In 16 out of 18 countries with an educational institution, the chiropractic education was accredited by an international chiropractic accreditation counsel or national government and/or federal institution [4].

As suggested by Meeker and Haldeman in 2002 [3] the future role of the profession was dependent on its ability to commit to interdisciplinary cooperation and science-based practice. However, despite an international movement towards a scientifically active, evidence-based profession, integrated in the public health systems, a large proportion of the profession do not adhere to advertising guidelines on their websites [6] and still offers treatment of non-musculoskeletal problems [7].

In Denmark, the government funded national Health Insurance has provided partially reimbursed chiropractic treatment since 1978 and licensing laws have been in place since 1992 [8–10]. A five-year master’s degree programme leading to chiropractic licensing was established in 1994, and is integrated with the medical faculty at a large publicly funded university [11]. As such, the education is firmly rooted in health sciences, with a high scholarly/academic profile. According to the Danish Authorisation Act, the chiropractic scope of practice includes diagnostics, prevention and treatment of biomechanical disorders of the spine, pelvis and extremities and does not mention non-musculoskeletal disorders [10]. Therefore, both the official governmental view and the self-perception of Danish chiropractors is that of a profession specialized in management of musculoskeletal disorders.

This study aimed to investigate the content and characteristics of website claims by chiropractors in Denmark on non-musculoskeletal disorders and to assess whether these were aligned with the legislated scope of practice.

The specific objectives were to investigate i) the type and extent of non-musculoskeletal diagnostic categories for which treatment is offered according to chiropractic websites and ii) whether the geographic position, size of the clinic, educational background of the chiropractors, coverage by government health scheme as well as advertising aiming for specific patient groups were associated with the content of the websites.

## Methods

### Design

An observational cross-sectional study.

### Setting

Data were collected from websites owned by chiropractic clinics in Denmark. A list of all chiropractic clinics in Denmark was retrieved from a public website [12] owned and operated by the Danish Health Authorities. The website of the Danish Health Authorities contains contact information on all chiropractic clinics in Denmark, including information about public reimbursement. A Google search was performed to locate websites related to all the recorded chiropractic clinics to ensure that only clinics with a current website were included.

Due to practical reasons only a sample representing approximately half of the chiropractic websites in Denmark could be assessed. To make sure that all five regions in Denmark were represented equally, 30 websites per region were sampled randomly, resulting in a maximum of 150 sampled websites. It was recorded whether the clinic was part of the financial reimbursement agreement negotiated between the regional Danish Health Authorities and the Danish Chiropractic Association. As almost 90% of chiropractic clinics in Denmark were part of the governmental partial reimbursement agreement [12], the clinics were selected 4:1 with and without agreement, respectively, to ensure a sufficient number of clinics without reimbursement agreement. The clinics were randomly selected using Googles ‘Random number generator’ [13] and the sampling was stratified with regards to geographic location and public reimbursement status.

### Data collection

Prior to data collection, five clinics from each region (25 in total) were randomly selected and diagnostic categories were collected to develop a data collection tool (Additional file 1). Using the data collection tool, two of the authors (HN/MA) independently examined the sampled websites and extracted data between December 20, 2018 and March 29, 2019. In case of disagreement, consensus was reached by discussion. Websites reporting non-musculoskeletal conditions were revisited by a senior researcher (RKJ) in December 2019 to update possible changes, with regards to any clarifying explanations provided.

To ensure that all relevant information was retrieved new variables were added if they emerged during data collection. Website information was recorded as present if mentioned on the website in the main text or in a drop-down menu. If the chiropractic clinic website provided a link to another website describing a disorder it was recorded as not present unless the chiropractic website clearly mentioned that the link provided information on the conditions treated in the clinic.

### Variables of interest

#### Clinics

The geographical location of a clinic and whether the clinic was acceded to the reimbursement agreement was recorded before the selection of clinics.

To enable an equally distributed geographical representation the region of each clinical was recorded (1) The North Denmark Region, 2) Central Denmark Region, 3) The Region of Southern Denmark, 4) Region Zealand or 5) The Capital Region of Denmark).

Country of education of the chiropractor was categorised as ‘Denmark’, ‘USA’ and ‘UK’, the three countries where the majority of Danish chiropractors are educated. If the chiropractor was educated in another country, this was categorised as ‘Other’. Clinics were categorised as ‘Danish’ if all chiropractors working in the clinic were educated in Denmark, ‘USA’ if all chiropractors were educated in USA etc. If the chiropractors working in the clinic had a mixed educational background (e.g. one from Denmark and one from USA) the clinic was recorded as ‘Mixed education’. If data were not available, it was recorded as missing.

Clinic size was defined as the total number of chiropractors working in the clinic independent of other professions working in the same clinic e.g. physiotherapists, medical doctors, acupunctures, massage therapist, reflexology, craniosacral therapist etc. The clinic was categorised as small if there was only one chiropractor and large if more than one chiropractor worked in the clinic.

#### Official chiropractic website

Kiropraktorguide.dk [14] (‘Guide to chiropractic’) is a website owned and operated by The Danish Chiropractic Association to provide patients with information about the conditions that chiropractors treat. In addition, patients can find information about most chiropractic clinics in Denmark and find a chiropractor in the area where they live. Kiropraktorguide.dk was created as a communal effort involving approximately 80% of Danish Chiropractors to provide a shared, central site of information of relevance to chiropractic patients.

The website provides useful information for patients and it was therefore recorded whether the individual clinic websites provided a link to Kiropraktorguide.dk.

#### Patient groups

Some chiropractors advertise that they have a special interest in treating specific patient groups. This information was recorded and categorised as ‘infants’, ‘children’, ‘seniors’, ‘pregnant women’, ‘athletes’ and ‘disabled people’.

#### Diagnosis and symptoms of non-musculoskeletal conditions

Information on non-musculoskeletal conditions from the websites was noted in the data collection tool. (See Additional file 1) If a diagnosis or symptom did not fit any of the predefined categories a new category was added. Predefined categories included both specific diagnosis such as ‘High blood pressure’ or ‘Allergy’ and more general symptoms as for example ‘Abdominal pain’.

For each non-musculoskeletal condition it was recorded whether a clarifying explanation was available and what it said. These explanations were assessed and categorised for compliance with the chiropractic scope of practice as defined by the Danish Authorisation Act.

In case of disagreement between the two data collectors, agreement was reached by discussion or by consulting a third author (RKJ).

The clarifying explanations were scrutinized to clarify, whether the websites actually claimed to offer treatment for non-musculoskeletal conditions, as opposed to describing non-musculoskeletal symptoms as secondary to musculoskeletal disorders or vice versa. Examples of adequate and inadequate explanations are presented in Additional file 2.

### Statistical analysis

The results are presented as descriptive statistics and frequency tables.

Differences in characteristics of the clinics and the frequency of reporting treatment of non-musculoskeletal conditions were tested using Pearson’s chi-squared test. When subgroup differences were significant, pairwise (post-hoc) comparisons were performed using chi-square tests for proportions or Fishers exact test to identify the specific groups that differed. Statistical significance level was set at 5%. STATA 16 (Stata Corp, College Station, Texas, USA) was used for analysis and data management.

## Results

**Fig. 1:**
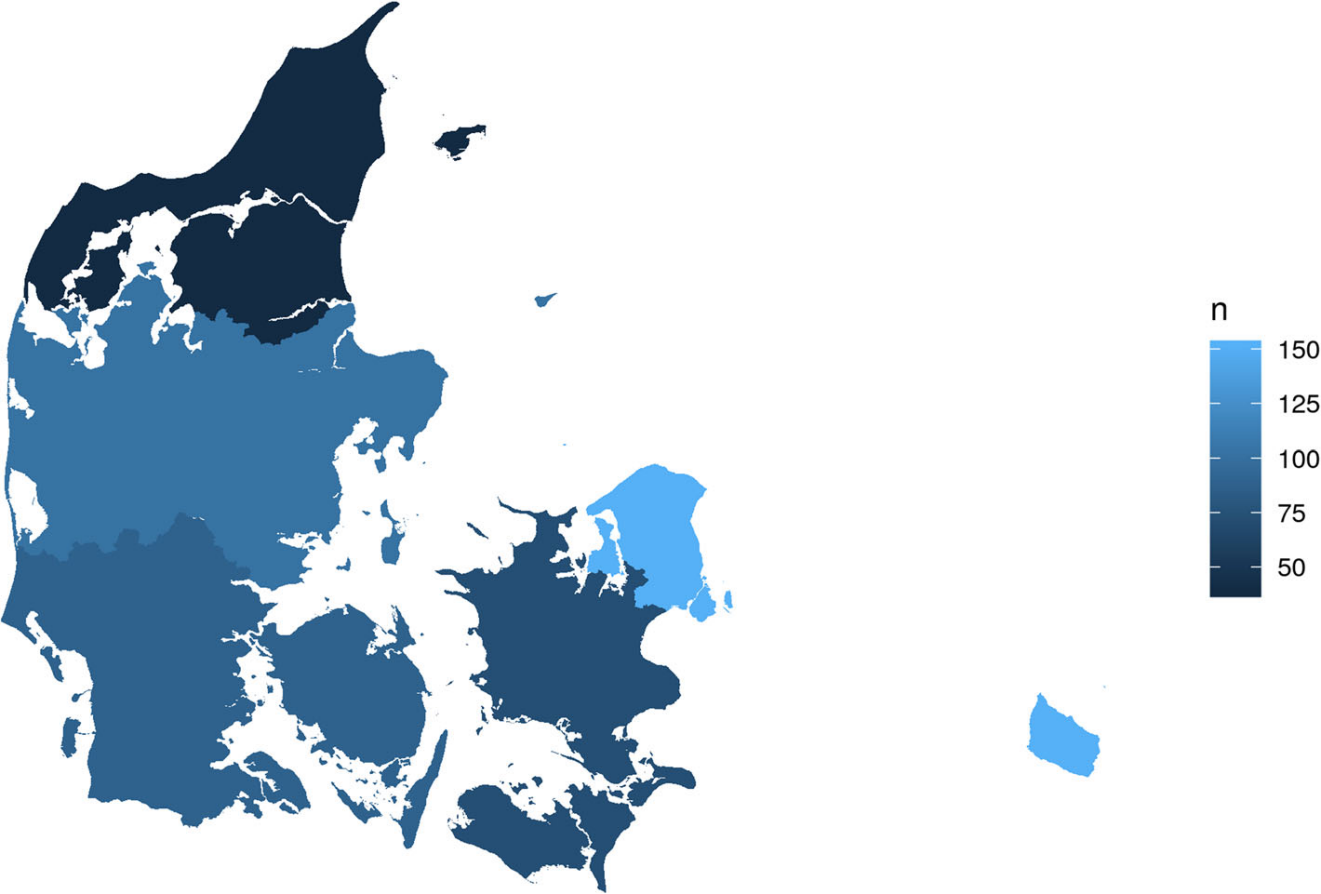
The distribution of clinics by Regions in Denmark

A total of 264 chiropractic clinics were registered in Denmark at the time of data collection. Of these 243 had a website. A sample of 30 websites per region were planned but as The North Denmark Region only had 20 chiropractic clinics, a total of 140 websites were assessed. One website was a duplicate as the clinic had two postal addresses and one was excluded. Finally, a total of 139 websites were included. The distribution of clinics throughout the country is shown in Fig. 1. This random sample represented 57% of the websites from chiropractic clinics in Denmark. Of these, 116 had a reimbursement agreement and 23 did not. The data are available in Additional file 3.

### Clinics

In 53 of the clinics, all the chiropractors were either educated abroad (UK *n* = 14 or USA *n* = 15) or in Denmark (*n* = 24), whereas the remaining 86 clinics had chiropractors with a mixed educational background (*n* = 42) or the information was not available on the website (*n* = 44).

Of the 139 clinics 35% were categorised as small (1 chiropractor) and 65% were large (≥2 chiropractors).

The website Kiropraktorguide.dk (‘Guide to chiropractic’), which describe what chiropractic is and where to find a chiropractor in the local area was referenced by 13% of the 139 websites.

### Patient groups

Infants (81%) and children (81%) were the patient groups most often mentioned on the websites as a special interest area, with 109 clinics (78%) mentioning both groups. Chiropractic care for senior citizens was found on 47%, pregnant women on 49%, athletes on 58% and disabled people on 3% of the websites.

### Diagnosis and symptoms of non-musculoskeletal conditions

**Fig. 2:**
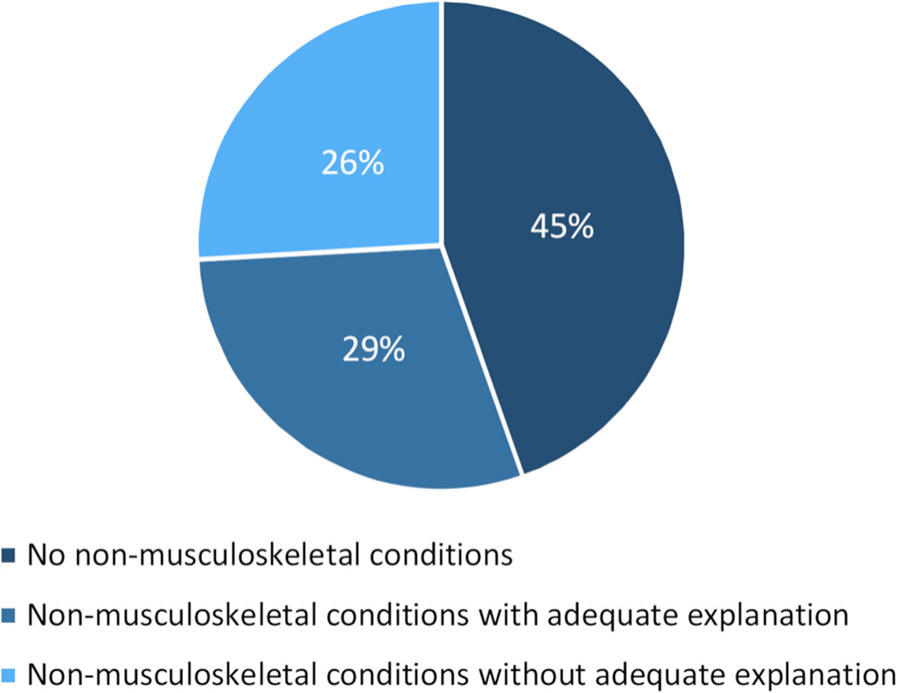
The proportion of chiropractic websites reporting non-musculoskeletal conditions with or without clarifying explanations

A total of 42 different diagnoses or symptoms of non-musculoskeletal origin were identified on 77 (55%) of the 139 websites. Of these,41 (53%) websites provided an adequate clarifying explanation and thus did not claim to be treating non-musculoskeletal disorders. See Fig. 2. 36 (26%) websites claimed that chiropractic services were offered for one or more of 26 different non-musculoskeletal conditions. Of these 36 websites, 26 (72%) mentioned 1–2 non-musculoskeletal conditions, six (17%) mentioned 3–5 different conditions, three (8%) mentioned 6–10 conditions and one (3%) website mentioned more than 10 different non-musculoskeletal conditions.

The non-musculoskeletal conditions most often mentioned on the websites were “Concentration problems (in children)” (*n* = 13)(36%), “Insomnia/unease/discontent (in children)” (n = 13)(36%), “Otitis media (in children)” (*n* = 11)(31%), “Constipation/digestive problems” (*n* = 8)(22%), “Abdominal pain” (*n* = 6)(17%), “Incontinence/bed-wetting/enuresis (in children)” (n = 6)(17%), “Problems with suckling/breast-feeding (in children)” (*n* = 5)(14%), “Hyperactivity/restlessness (in children)” (*n* = 4)(11%), whereas the rest of the conditions were mentioned by less than 4 of the websites. Nine of the 26 (25%) different non-musculoskeletal conditions concerned children. See Table 1.

**Table 1:** Non-musculoskeletal diagnosis and symptoms reported on 139 Danish websites

Symptom/diagnosis		Number of clinics reporting non-MSK in total (*n* = 77)	Number of clinics reporting non-MSK without an adequate explanation (*n* = 36)
1	Concentration/attention problems (in children)	25	13
2	Insomnia/unease/discontent (in children)	43	13
3	Otitis media/ear infection (in children)	24	11
4	Constipation/digestive problems	17	8
5	Abdominal pain	20	6
6	Incontinence/bed-wetting (in children)	13	6
7	Problems with suckling/breast-feeding (in children)	19	5
8	Hyperactivity/ restlessness (in children)	12	4
9	Asthma	15	3
10	Learning problem (in children)	9	3
11	Tinnitus	3	3
12	Changes in mood (in children)	5	2
13	Infection	4	2
14	Menstrual cramps/pains	2	2
15	Respiratory problems (other than asthma)	4	2
16	Vestibular neuronitis	7	2
17	Allergy	4	1
18	Attention-deficit/hyperreactive disorder (in children)	1	1
19	Common cold	1	1
20	Concussion	5	1
21	Eye and ear pain	1	1
22	Hormonal imbalance	1	1
23	Immune system	2	1
24	Language, reading or writing difficulties	5	1
25	Nausea	2	1
26	Sinusitis	2	1
27	Chronic fatigue syndrome (CFS)	1	0
28	Chronic Obstructive Pulmonary Disease	2	0
29	Complex regional pain syndrome (Type I)	2	0
30	High blood pressure	2	0
31	Highly sensitive children	3	0
32	Impotence	2	0
33	Internal organs	4	0
34	Irritable bowel syndrome	1	0
35	Low blood pressure	2	0
36	Ménière’s disease	2	0
37	Osteoporosis	4	0
38	Shingles (herpes zoster)	1	0
39	Swelling/ bleeding/wounds	2	0
40	Trigeminal neuralgia	1	0
41	Tumour	2	0
42	Vision impairment/disturbance	1	0

### Differences between clinics

**Fig. 3:**
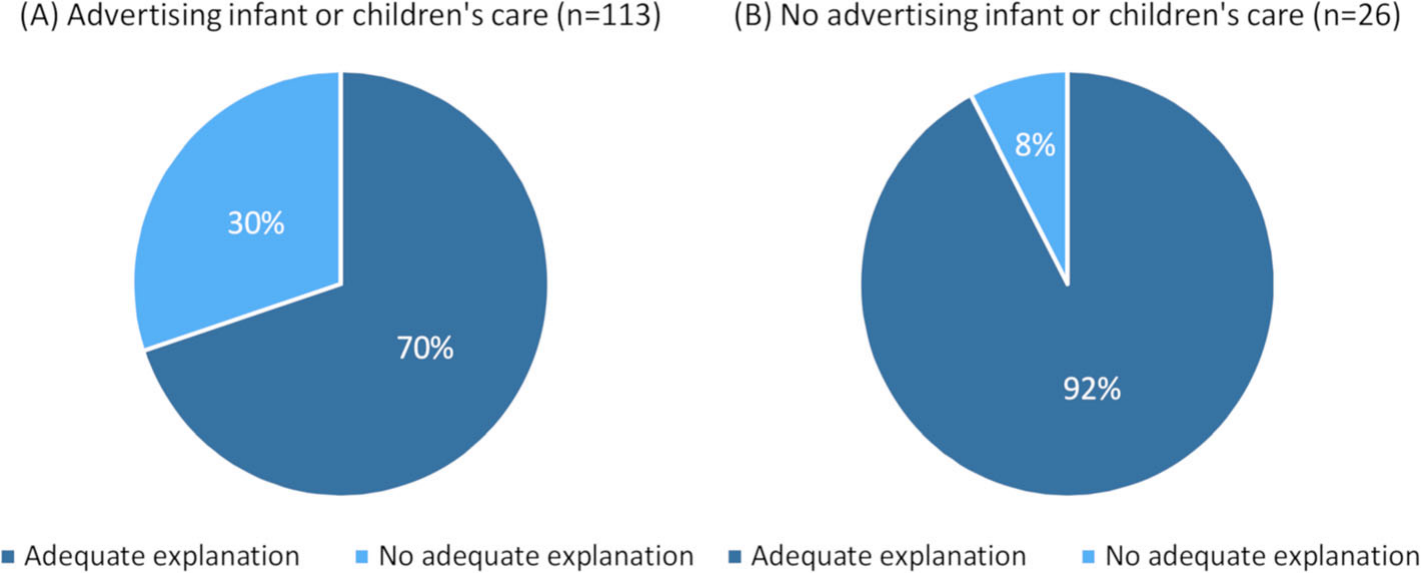
Chiropractic websites advertising infant or children’s care **a** or not **b** in relation to claims of non-musculoskeletal conditions

No difference in location, clinic size, reimbursement status or country of educational background was observed between those clinics which advertised treatment for non-musculoskeletal disorders and those which did not. However, there was a larger proportion of websites advertising treatment for non-musculoskeletal disorders without providing an adequate explanation among clinics with a special clinical focus in infants/children (31%) compared to websites that did not focus on infants/children (7%) (*p* = 0.005). See Fig. 3.

## Discussion

Of the 139 (57%) chiropractic clinic websites audited in Denmark, 36 (26%) mentioned diagnoses or symptoms of non-musculoskeletal origin that was not accompanied by a clarifying explanation in agreement with the chiropractic scope of practice as defined by the Danish Authorisation Act. Most of these clinics (72%) mentioned one or two different non-musculoskeletal conditions which were most often related to “Concentration / attention problems (in children)”, “Insomnia/unease/discontent (in children)” or “Otitis media / ear infection (in children)”. Six of the eight non-musculoskeletal conditions mentioned by more than three websites were related to children.

In a study by Murdoch et al. [15] 100 chiropractic websites in Canada were examined and 33% claimed diagnosis, treatment and/or efficacy for allergy/sensitivity and 38% for asthma. In comparison, allergy was mentioned on four out of 139 websites (3%) in the present study and only one website (< 1%) did not provide an adequate explanation. By contrast, asthma was mentioned on 15 out of 139 websites (11%) and only three (2%) did not provide an adequate explanation. At face-value, this suggest that a higher proportion of Canadian chiropractors advertise treatment for non-musculoskeletal disorders, compared to the Danish sample. However, in the study by Murdoch et al. an electronic search tool was used to detect key words mentioned on the websites. Therefore, it is possible that the Canadian results included websites reporting that they offered treatment for musculoskeletal pain secondary to for example asthma. However, even when all mentioning of allergy or asthma in our study is considered, the numbers seem to be considerably higher in the Canadian study.

A study by Ernst and Gilbey [16] from 2010 investigated the frequency of claims to treat conditions not supported by evidence such as asthma, migraine, infant colic, colic and ear infection/earache/otitis media by chiropractors in the English-speaking world. The study found that among 200 chiropractic websites 95% made unsubstantiated claims. In the study by Ernst and Gilbey [16] 52% reported treating asthma compared to 11% in our study (2% without an adequate explanation) and 55% reported treating ear infection/earache/otitis media compared to 17% in our study (9% without an adequate explanation). In general, Danish chiropractic websites seems to provide fewer claims about treatment for non-musculoskeletal conditions.

In our study, there was a positive association between clinics advertising treatment specifically for infants or children and the mentioning of one or more non-musculoskeletal conditions without an adequate explanation. Forty-three (39%) websites advertising infant or children’s care mentioned one or more non-MSK conditions without adequate explanation. Also, 35% of the non-musculoskeletal diagnoses and symptoms that were mentioned without an adequate explanation were related to infants or children. In a study by Cherkin et al. from 2002 [17] describing patients and problems seen by complementary and alternative medical practitioners, 3–4% of patients seeking a chiropractor were < 15 years of age and the contact reason was mainly related to musculoskeletal conditions. According to Statistics Denmark [18] a comparable 3.4% of visits to a chiropractor in Denmark in 2006 were by persons < 15 years. However, in 2018 the number had increased to 6% primarily due to a rise in infants (0–1 year) seen by chiropractors. There could be an association between the increase in child patients and the high number of non-musculoskeletal conditions advertised on websites. This is supported by a recent study by Marcon et al. (2019) [19] who found a trend for marketing of chiropractic for children (8%) in a study of chiropractic 369 websites from clinics in Canada.

Danish chiropractors do not advertise treatment of non-musculoskeletal conditions as often as chiropractors in other countries as reported by other studies [15, 16]. Nonetheless, as the Danish chiropractic profession enjoys an overall high level of integration in the public health care system and the chiropractic scope of practice is clearly set out in legislation, 26% is a surprisingly high percentage.

### Strengths and weaknesses of the study

The method used for data collection was a manual reading of the websites leading to risk of missing one or more diagnosis and symptoms. Also, there was a great deal of subjectivity when deciding if a non-musculoskeletal diagnosis or symptom was provided with an adequate explanation. However, two authors went through all websites and discussed disagreements, thus lowering the risk of bias and error. Furthermore, the numbers of non-musculoskeletal diagnosis and symptoms both with and without adequate explanations are reported for transparency. The difference in proportions of non-musculoskeletal conditions without a clarifying explanation between clinics advertising infant or children’s care should be interpreted with caution due to low cell sizes.

Despite scrutinising the online descriptions, it is possible that some websites were identified as promoting chiropractic care for non-musculoskeletal disorders, when in fact they simply failed to communicate adequately, that in some instances musculoskeletal symptoms can be misinterpreted as originating in other organs. Similar considerations may apply to the previously mentioned studies from Murdoch et al. and Ernst & Gilbay. Therefore, further research should focus on qualitative studies interviewing the chiropractor responsible for the websites to explore the intensions and meaning of the website content. Imprecise communication may lead to confused or misinformed patients and in worst case delay correct treatment for a non-musculoskeletal condition. Also, the Danish chiropractic profession works closely with other health care professions and is an integrated part of the Danish health care system. Therefore, a common language and understanding of the biopsychosocial disease model is essential to ensure professional communication with the health care system to the benefit of the patients.

## Conclusions

In this sample, 36 (26%) chiropractic clinics in Denmark mentioned diagnoses or symptoms of non-musculoskeletal origin on their websites without providing an adequate clarification. It is unclear to what extent this reflects a subgroup of chiropractors inclined to offer treatment for non-musculoskeletal disorders, or a subgroup of websites which fail to communicate adequately. The former, would run counter to the scientific literature and the chiropractic scope of practice as defined by the Danish Authorisation Act, and thus misdirect patients. The latter could be misleading for patients seeking care for non-musculoskeletal conditions. Further research is recommended to clarify this.

## Supplementary information

**Additional file 1.** Data collection tool.

**Additional file 2.** Examples of adequate and inadequate explanations from websites.

**Additional file 3.** Copy of dataset.

## Data Availability

All data generated or analysed during this study are included in this published article in Additional file 3.xls.

## Abbreviations

CFS: Chronic fatigue syndrome
MSK: Musculoskeletal
UK: United Kingdom
USA: United States of America
