# Supplementary material for: A cross-sectional study of website claims related to diagnoses and treatment of non-musculoskeletal conditions
Source: Chiropr Man Therap. 2020 Mar 30;28:16. doi: 10.1186/s12998-020-00305-w (PMC7106824; doi:10.1186/s12998-020-00305-w)
Supplement: Supplementary file 1 — Additional file 1. Data collection tool. [file 12998_2020_305_MOESM1_ESM.pdf]

Additional file 1

Data collection tool for non-musculoskeletal symptoms and diagnosis

Original data collection tool developed from 25 websites	Additional symptoms and diagnosis added	Present = 1<br>Not present = 0	MSK based explanation
Abdominal pain			
Allergy			
Asthma			
	Attention-deficit/hyperreactive disorder (in children)		
	Changes in mood (in children)		
Chronic fatigue syndrome (CFS)			
Chronic Obstructive Pulmonary Disease			
	Common cold		
	Complex regional pain syndrome (Type 1)		
Concentration problems (in children)			
	Concussion		
Constipation/digestive problems			
	Eye and ear pain		
	High blood pressure		
	Highly sensitive children		
	Hormonal imbalance		
	Hyperactivity/restlessness (in children)		
	Immune system		
	Impotence		
	Incontinence/bed-wetting (in children)		
	Infection		
Insomnia/unease/discontent (in children)			
Internal organs			
Irritable bowel syndrome			
	Language, reading or writing difficulties		
Learning problem (in children)			
Low blood pressure			
Ménière's disease			
	Menstrual cramps/pains		
	Nausea		
	Osteoporosis		
Otitis media/ear infection (in children)			

	Problems with suckling/breast-feeding (in children)		
Respiratory problems (other than asthma)			
	Shingles (herpes zoster)		
	Sinusitis		
	Swelling/ bleeding/wound		
	Tinnitus		
	Trigeminal neuralgia		
	Tumour		
Vestibular neuronitis			
	Vision impairment/disturbance		
