# Supplementary material for: A cross-sectional study of website claims related to diagnoses and treatment of non-musculoskeletal conditions
Source: Chiropr Man Therap. 2020 Mar 30;28:16. doi: 10.1186/s12998-020-00305-w (PMC7106824; doi:10.1186/s12998-020-00305-w)
Supplement: Supplementary file 2 — Additional file 2. Examples of adequate and inadequate explanations from websites. [file 12998_2020_305_MOESM2_ESM.docx]

Additional file 2

# Examples of adequate and inadequate explanations of non-musculoskeletal symptoms and diagnosis

For each non-musculoskeletal condition, it was recorded whether a clarifying explanation was available and what it said. These explanations were assessed and categorised for compliance with the chiropractic scope of practice as defined by the Danish Authorisation Act.

The clarifying explanations were scrutinized to clarify, whether the websites actually claimed that treatment for non-musculoskeletal conditions was offered, as opposed to describing non-musculoskeletal symptoms as secondary to musculoskeletal disorders or vice versa.

## Internal organs

This is an example of an **adequate explanation** from a website [informal translation from Danish]:

“Chiropractors treat disabilities in the musculoskeletal system and the possible symptoms of the disabilities. Sometimes pain experienced as coming from the heart, lungs, stomach, biliary tract or other internal organs can originate from problems with the musculoskeletal system and can therefore be relieved by chiropractic treatment. Conversely, you can get back pain due to organic diseases such as gallstone or kidney stone as they can result in muscle tension. When an organic problem has been treated by a medical doctor, chiropractic can help get rid of the remaining back pain.” [1]

This is an example of an **inadequate explanation** from a website [informal translation from Danish]:

“The connection between the nervous system and the vertebrae of the spine is the reason why we help patients with headache, high blood pressure, low blood pressure, impaired lung function, impaired immune system, stomach discomfort etc. These are all disorders which can be caused by disrupted communication between the brain and organs.”[2]

## Insomnia/unrest/discontent (in children)

This is an example of an **adequate explanation** from a website [informal translation from Danish]:

“As well as adults, children may have problems with joints and muscles. However, in infants the symptoms are expressed in completely different ways. Typical symptoms of joint and muscle problems in infants can be e.g. insomnia, unrest, discontent…”. In this example, the text explains that symptoms such as insomnia and discontent in infants can be a symptom caused by musculoskeletal disorders. [3]

This is an example of an **inadequate explanation** from a website [informal translation from Danish]:

“A large proportion of our patients are children. XX in particular has acquired substantial expertise in both treatment and diagnostics of this unique group of patients. In infants crying due to colic, unease, poor sleep, uneven head shape, favorite side, breast preference and asymmetry (C-shaped) when lying down may indicate that something is not working properly.” In this example there is no explanation that the symptoms could be related to musculoskeletal pain. [4]

## Otitis media / ear infection (in children)

These are two examples of **adequate explanations** from two different website [informal translation from Danish]:

“Children: In slightly older children, we see crick in the neck, neck pain or back pain and perhaps headache as a result of dysfunctional joints.

Dysfunctional joints can also appear as symptoms similar to an ear infection. This is because neck tension can prevent the naturally occurring fluid in the middle ear from draining through the eustachian tube to the throat area. This leads to increased fluid in the middle ear, which can be painful.” [5]

“Earache, including otitis media, is frequent in infants and children. The pain often appears at night when the child is lying down. The child may be restless, rub the ear and may have fever. If you suspect that you child suffers from otitis media, you should of course consult your general practitioner for treatment of your child.

Ear problems in children can occur due to blocking in the tube (the eustatic tube) which connects the pharynx with the middle ear. Children's eustachian tubes lie more horizontally than they do in adults and are therefore more difficult to drain. Therefore, a blocking is often seen with, for example, mucous and/or pus, typically in relation to common cold. This blocking increases pressure in the ear and causes pain.

If your child experiences recurring ear problems, it may be because the neck does not move entirely as it is supposed to. Neck mobility helps to stimulate the eustatic tube for easier draining - but in case of dysfunctional joints and/or tension in the muscles in the neck, it can increase the risk of blocking in the middle ear.

Therefore, it may be a good idea to visit a chiropractor who can examine your child to find out if this is the case. If so, it can easily be treated.” [6]

This is an example of an **inadequate explanation** from a website [informal translation from Danish]:

“Treatment in babies and children: At XX Chiropractor Center, we treat a lot of children and babies every year. We treat a wide variety of things including colic, KISS/KIDD [Kinematic Imbalance due to Suboccipital Strain/ Kinematically Induced Dysgnosia and Dyspraxia], insomnia/unrest, otitis media, acute crick in the neck and much more. The treatment is often carried out with the child lying on the stomach of the mom or dad to make the child feel safe. It is very gentle and carried out quickly as children respond rapidly and effectively to chiropractic.” [7]

1. Als Kiropraktor Center. [cited 2019 December]; Available from: <https://dinkiropraktor.dk/behandlinger/>.

2. Stengadeklinikken, Kiropraktik & nervesystemet. [cited 2019 December]; Available from: <http://www.stengadeklinikken.dk/kiropraktor-i-aalborg/nervesystemet/>.

3. Kiropraktisk Klinik Vejen. [cited 2019 December]; Available from: <https://vejenkiropraktor.dk/spaedborn>.

4. Mariagerfjord Kiropraktorerne. [cited 2019 December]; Available from: <http://www.mariagerfjordkiropraktorerne.dk/praktisk/boern.html>.

5. Ida & Knud. [cited 2019 December]; Available from: <http://www.idaogknud.dk/>.

6. Vangede Kiropraktik & Sundhed. [cited 2019 December]; Available from: <https://vangedekiropraktik.dk/hvadbehandles/sp%C3%A6db%C3%B8rn/%C3%B8reb%C3%B8rn.html>.

7. Herlev Kiropraktor Center. [cited 2019 December]; Available from: <https://www.kirocenter.dk/om-behandlingen/baby-barn.aspx>.
